# Emergency Use of Tocilizumab in Moderate and Severe COVID-19 Patients over the Prevaccination Stage during the Pandemic in Mexico

**DOI:** 10.1155/2022/6880405

**Published:** 2022-12-06

**Authors:** Yessica Bernal Luna, Alejandro Esquivel Loza, José de Jesús Garduño García

**Affiliations:** ^1^Instituto Mexicano del Seguro Social Hospital General Regional 251, Metepec, Mexico; ^2^Universidad Autónoma del Estado de México, Facultad de Medicina, Toluca, Mexico

## Abstract

**Introduction:**

COVID-19 has been associated with the overactivation of the immune system; interleukin-6 (IL-6) seems to have a key role, which made its moderation to be proposed as a therapeutic blank. Tocilizumab has been used as part of treatments to modulate the immune response to infections caused by COVID-19. *Material and Methods*. An observational, descriptive, retrospective, and transversal study was carried out in patients diagnosed with moderate-severe COVID-19 who were hospitalized in the 251 General Regional Hospital from March to December 2020.

**Results:**

A review of 700 files corresponding to hospitalized patients was carried out, and a sample of 70 patients who met the inclusion criteria proposed for this protocol was obtained. Among the comorbidities associated with the disease, it was found that hypertensive patients have a higher mortality rate: 62% died and so did 59% of those who needed invasive respiratory support. As regards admission tests, statistical significance was found for the figures of leukocytes, neutrophils, glomerular filtration rate, and PCR.

**Conclusion:**

The use of this drug benefits mainly young nonhypertensive patients with a moderate disease and preserved renal functions with no need for invasive respiratory support, regardless of other comorbidities.

## 1. Introduction

In December 2019, the World Health Organization received a report from Chinese authorities regarding a group of 27 pneumonia cases with unknown etiology in the city of Wuhan (Hubei province) [1, 2].

As of November 9^th^, 2022, at the global level, 630,387,858 cases have been verified, while 6,583,163 deaths have been recorded. In America, 180,429, 028 cases have been confirmed, while in Mexico, the figures are 5,517 cases per 100,000 inhabitants and 256.6 confirmed deaths per 100,000 [3]. Up to the date mentioned above, 7,113,429 confirmed cases and 344,384 deaths have been registered in the country. In the state of Mexico, there have been 703,712 verified cases [4].

Several drugs have been put forward as candidate*s* for the treatment of this disease. Among them, we find those that block the bond of the host cell by means of angiotensin-converting enzyme (ACE), the union with TMPRSS2, endocytosis, viral replication, and inflammatory processes [5]. It is worth mentioning that none of the drugs mentioned above have been tried; however, they have been used as rescue treatments for this disease [6].

COVID-19 pneumonia has been associated with lung damage, severe immunosuppression, and lymphopenia. In this scenario, the presence of some immunological biomarkers in the prognosis of the disease has been assessed, as demonstrated in a prospective study, which reported a worse prognosis in patients with ANA autoantibodies, reinforcing the role of inappropriate immune regulation in this disease [7]. On the other hand, there are cases of autoimmune thrombocytopenia associated with COVID-19, which support the presence of alterations in immune regulation [8].

In Iraq, a cross-sectional study was performed on COVID-19 patients for the purpose of finding out the correlation among routine hematological, immunological, and inflammatory markers, as well as their relationship with the severity of the disease. In this study, the authors found out that the IL-6 count of lymphocytes and neutrophils represents major risk factors in the severity of this disease [9].

Since an increase in the levels of IL-6 has been described as a consequence of the overactivation of the immune system in COVID-19 patients, the use of a blocker for such interleukin was suggested in order to limit the inflammatory process triggered by the virus [10–12].

Blocking IL-6 by means of an antibody against its receptor has proven to be efficacious in various autoimmune diseases. Moreover, the use of the recombinant monoclonal antibody receptor of antihuman IL-6, tocilizumab, is presently approved for rheumatic diseases such as rheumatoid arthritis and juvenile idiopathic arthritis [13, 14].

There is limited information regarding the use of this drug in COVID-19 patients, mainly from retrospective studies with few patients. Also, there are limited data about the outcomes of using this drug in Latin America and Mexico. The few existing trials are limited to critical disease conditions in specialized medical centers.

The following study describes a retrospective analysis on the characteristics and outcomes of patients who received tocilizumab during the first stage before the COVID-19 vaccination campaign started in Mexico, in a second-level hospital, as a rescue treatment for moderate to severe disease. The purpose of this characterization was to assess the variables related to prognosis improvements and mortality diminution in Mexican patients.

## 2. Methodology

This is a single-center, observational, retrospective, and transversal study carried out at Hospital General Regional No. 251 (HGR 251) of the Mexican Social Security Institute (IMSS).

The sample comprised patients older than 18 years of age with a diagnosis of moderate to severe COVID-19 who were hospitalized in the facilities mentioned above during the first and second waves of COVID-19 before the vaccination campaign.

The inclusion criteria considered patients who had previously received tocilizumab doses in the internal medicine hospitalization service according to the internal algorithms of IMSS [15], while the exclusion criteria screened patients with contraindications due to tocilizumab hypersensitivity, latent tuberculosis, a history of diverticulitis, and verified bacterial infections associated.

### 2.1. Statistical Analysis

The results were recorded in a digital database. Qualitative values will be expressed as means with ranges and standard deviation, while qualitative, in percentage. The Kolmogorov–Smirnov (KS) test was applied to define normality. Parametric quantitative values were analyzed with a Student's *T*-test, whereas the nonparametric quantitative values were analyzed with Mann–Whitney U test. Finally, qualitative variables were analyzed by means of the chi-squared test. Statistical analyses were run using Statistical Software for Social Sciences (IBM SPSS Statistics for Windows, Version 22.0, Armonk, NY: IBM Corp).

### 2.2. Ethical Aspects

Since the research proposal corresponds to a study with no risk and retrospective in nature, permission was asked to avoid using an informed consent letter in the understanding of the obligation of preserving the confidentiality of personal and medical data and also restating the commitment to only obtaining the information necessary for the present study, which will be used in the research protocol.

The study was submitted to the local research committee for revision, and after meeting the ethical requirements for research, it was approved with the registration number R-2021-1503-049.

## 3. Results

A review of the files of 700 patients admitted to Hospital General Regional 251 during the first and second COVID-19 waves, before the beginning of the vaccination campaign, was carried out, and a sample of 70 patients was obtained. The patients meet the inclusion criteria proposed for this protocol. Among the basal characteristics of the population, we have a mean age of 49 years, ranging from 23 to 78 years; men account for 58.6% of the sample. The mean of the onset of symptomatology was 8.49 ± 6.91 days. Obesity, hypertension, and diabetes mellitus were found among the most prevalent comorbidities; other basal characteristics are displayed in Table 1.

In addition to the treatment with tocilizumab, the totality of patients received enoxaparin, 94.3% clarithromycin, and 70% steroids.

Among the comorbidities associated with the disease, it was found that hypertensive patients have a higher mortality rate; 62% of these patients died, and no other comorbidity was statistically significant for the outcome of patients who received the drug; other comorbidities and their related mortality are displayed in Table 2.

Furthermore, 59% of the patients who needed invasive respiratory support died; this implies a worse prognosis for patients who received the drug and were intubated.

As for the pharmacological treatment associated with tocilizumab use and hospital outcome, we did not find statistical significance for any of the drugs mentioned above.

It was noticed that at younger ages, there is a higher likelihood for survival, considering that the average age of the survivors was 47.6 years, as compared with 54.45 of those who died.

In relation to admission tests, statistical significance was found for leukocytes, neutrophils, glomerular filtration rate (GFR), and PCR. While there was a higher count of leukocytes and neutrophils, there was an increase in mortality.

Renal function deterioration, expressed as a diminution of GFR, is associated with a worse prognosis as compared with those who retain good renal function.

Higher levels of PCR were associated with higher mortality in patients who received the drug. Other admission tests and their related mortality are displayed in Table 3.

## 4. Discussion

The COVID-19 pandemic produced an unprecedented situation for which there were neither defined strategies nor guidance to treat this disease. In this context, various drugs for its treatment appeared [5, 16, 17] previously existing treatments were utilized, some of them indicated for chronic conditions [18]; among the drugs used to restrict the inflammatory process were found the antagonist of IL-6, also known as pleiotropic cytokine, produced by a large amount of hematopoietic and somatic cells, which are in charge of multiple biological activities [13, 14].

The receptor of IL-6 (IL-6R) has two forms: one joined to the receptor of the interleukin-6 membrane (mIL-6R) and another as a soluble receptor (sIL-6R). IL-6 joins mIL-6R to produce a complex that then joins the gp130 protein to induce intracellular dimerization and signaling of the kinase Janus pathway/transducers and activators of the transcription signal (JAK/STAT); moreover, the phosphatase SHP2 joins the phosphorylated gp130 and mediates the activation of the Ras-Raf-MAPK pathway [19, 20].

sIL-6R may be generated via two different mechanisms, i.e., the proteolysis of the membrane receptor by the metalloproteinase ADAM17 and the transition of spliced mRNA. Through this receptor, cells with the protein gp130 in their membrane may be activated by IL-6 even in the absence of its membrane receptor [20].

The activation of the cells by means of sIL-6R represents the proinflammatory activity of this interleukin, which leads to the recruitment of mononuclear cells, the inhibition of both T lymphocyte apoptosis and the differentiation of T-regulator lymphocytes, and the stimulation of endothelial cells for MPC1 production [20]. However, the activation of the membrane receptor implies the activation of the STAT3 pathway, which generates the proliferation of epithelial intestinal cells, inhibits apoptosis at the epithelial level, and also induces a hepatic response to inflammatory processes [20].

The blocking of IL-6 by means of an antibody against its receptor has proven to be efficacious in various autoimmune diseases [19]. Presently, tocilizumab is approved for use in rheumatic diseases such as rheumatoid arthritis and juvenile idiopathic arthritis [13, 14].

The use of this sort of drugs in the face of a scarcely studied situation has various implications, among which one finds the following: (1) the risk of not administering a drug that may improve the health of a patient positive to SARS-CoV2; (2) the adverse effects of using a nonapproved drug for this disease; and (3) the risk of harming patients with chronic pathologies who need constant administration of these drugs and did not find them due to their limited availability [18].

The scarce information about the use of this drug in patients with COVID-19 mainly comes from retrospective studies with few patients such as a single-center retrospective study in Italy, which included patients hospitalized in an intensive care unit. A total of 65 patients who met the inclusion criteria were selected. These criteria considered PCR-confirmed disease and the hyperinflammation stage, defined as PCR over 100 mg/L, ferritin over 900 ng/mL, LDH over 220 U/L, compatible tomographic data, and PaO_2_/FiO_2_ under 300. Patients were grouped to receive standard treatment, as compared with standard treatment plus tocilizumab 400 mg, in two doses, finding a higher survival and lower intubation rate in patients who were given this drug [21].

Another single-center retrospective cohort study carried out in Cleveland Clinic intended to find out the outcome of patients treated with tocilizumab; the sample comprised 51 patients, out of which 55% received this drug. In the analysis, patients who were given this drug experienced clinical improvement in shorter time (8 v. 13 days), used fewer vasopressors, and required less invasive mechanical ventilation [22].

A randomized open and controlled study was carried out in the United Kingdom to assess the various possible treatments for COVID-19 in such country and included patients with saturation under 92% or with supplementary oxygen requirements, as well as evidence of systemic inflammation, to randomize them in standard care compared with standard care plus tocilizumab. Among the findings of this study, we find a higher likelihood of hospital admission at 28 days, 57% v. 50%, with a lower possibility to undergo advanced respiratory tract management or end up in death, 35% v. 42%. In this way, it is concluded that this drug is able to improve survival and other clinical outcomes as well [23].

Some retrospective studies have demonstrated no diminution for mortality or during respiratory support in patients with severe disease [21, 22].

However, other studies, i.e., COVINTOC, an open-label, randomized, phase-3 trial that compared the use of tocilizumab supplied as a single infusion at 6 mg/kg v. standard care did not show any benefit [24].

A randomized, double-blind, placebo-controlled phase-3 trial evaluated the safety and efficacy of tocilizumab in hospitalized patients with COVID-19 pneumonia with no need for mechanical ventilation; the selection of patients focused on the inclusion of high-risk patients and the minority population, which comprises 56% of Hispanics or Latinos. The authors conclude that for hospitalized patients with COVID-19 pneumonia who were not receiving mechanical ventilation, tocilizumab reduced the likelihood of progression, nevertheless it did not improve survival [25].

In Mexico, there are few studies on this drug. One of them was a prospective cohort study that included 99 patients with severe and critical COVID-19 for the purpose of assessing the effect of tocilizumab on hospital mortality in severe and critical COVID-19 patients in a third-level medical center. The study found no differences between groups and mortality and concluded that tocilizumab was not associated with decreased hospital mortality [26].

Furthermore, the role of timing in the administration of this drug has been studied in a retrospective, observational study that was carried out with the aim of evaluating the mortality and prognosis of different administration times of tocilizumab, and it was found that mortality was only reduced when the drug was administered at early inflammatory stages (8 to 15 days of the symptoms) [27].

Some of the clinical characteristics associated with the outcomes after using tocilizumab in the present retrospective study are as follows: clinical response was favorable in younger patients; age was not described in other retrospective studies as a critical factor for the outcome of the use of this drug; however, being hypertensive was associated with an increment in mortality, while the rest of the comorbidity characteristics did not have a statistically significant impact on the outcome of the patients. In like manner, vital signs at admission did not make a significant difference in the outcome.

Patients with good renal functions were additionally benefitted by the use of tocilizumab as compared with those with deteriorated functions, still with no need for therapy to substitute renal functions.

Higher levels of C-reactive protein are associated with the worst outcome. Moreover, the vast majority of patients received other drugs in addition to tocilizumab, and this did not modify the end result; in any case, the need for invasive respiratory support was associated with higher mortality; as noticed in other trials, maybe the use of tocilizumab on patients with no need for mechanical ventilation is associated with the improvement of mortality rates.

## 5. Conclusion

We may conclude that the use of this drug mainly benefits young, nonhypertensive patients with a moderate disease, preserved renal functions, and no need for invasive mechanical ventilation, regardless of the rest of their comorbidities.

Among the limitations of the study, we have that it was carried out in a single center with a limited sample and no placebo comparison; plus, there was no analysis on invasive respiratory support. A study on a larger population is necessary for more effectively ascertaining the characteristics of the population for which this drug is useful.

## Figures and Tables

**Table 1:** Basal characteristics of the population.

Age (years)	49 ± 11.9
Man%	58.6
Woman%	41.4
Days from onset	8.49 ± 6.91
BMI kg/m^2^	29.2 ± 5.02

*Comorbidities*	
Asthma %	2.9
Diabetes mellitus %	21.4
COPD %	1.4
Obesity %	31.4
Hypertension %	22.9
CKD %	1.4
Smoking %	2.9

*Vital signs at admission*	
SaO_2_% without O_2_	73.46 ± 12.96
SaO_2_% with O_2_	87.09 ± 8.38
Heart rate	92 ± 16.66
Respiratory rate	23.9 ± 4.60

*CO-RADS %*	
3	8.6
4	32.9
5	58.6
Respiratory support %	31.4

*Test values*	
Leucocytes ×10^3^/uL	10−3 ± 4.70
Neutrophils ×10^3^/uL	9.0 ± 4.65
Lymphocytes ×10^3^/uL	0.85 ± 0.50
Glucose mg/dL	152 ± 103.09
ALT mg/dL	59.23 ± 82.77
AST g/dL	67.10 ± 128.50
LDH mg/dL	493.57 ± 286.22
Bilirubin total mg/dL	0.63 ± 0.35
CK mg/dl	109.91 ± 103.79
GFR	88.63 ± 30.22
Fibrinogen mg/dL	1358 ± 2451.69
PCR	20.60 (±11.74

*Other drug*s	
Hydroxychloroquine	11.4
Azithromycin	4.3
Clarithromycin	94.3
Enoxaparin	100
Oseltamivir	22.9
Steroids	70
Lopinavir/Ritonavir	15.7

**Table 2:** Comorbidities and mortality.

	Released alive	Decease	
Sex			*p*=0.952
Men	68% (28)	31% (13)	
Women	68% (20)	31% (9)	

Comorbidities			
Asthma	100% (2)	0% (0)	*p*=0.331
Diabetes mellitus	73% (11)	26% (4)	*p*=0.624
COPD	0% (0)	100% (1)	*p*=0.137
Obesity	72% (16)	27% (6)	*p*=0.612
Hypertension	37% (6)	62% (10)	*p*=0.002
CKD	0% (0)	100% (1)	*p*=0.137
Smoking	50% (1)	50% (1)	*p*=0.566

CO-RADS			*p*=0.168
CO-RADS 3	50% (3)	50% (3)	
CO-RADS 4	82% (19)	17% (4)	
CO-RADS 5	63% (26)	36% (15)	
Mechanical ventilation	40% (9)	59% (13)	*p*=0.001

**Table 3:** Mortality-related quantitative characteristics of the population.

Characteristic	Alive	Death	*p* value
Onset of the symptoms	8.25 ± 3.884	9 ± 4.493	0.47
Age	47.6 ± 11.32	54.45 ± 12.32	0.025
BMI	29.1 ± 4.91	29.43 ± 5.34	0.824
SaO2% without O_2_	74.33 ± 13.98	71.55 ± 10.34	0.40
SaO2% with O_2_	87.75 ± 9.39	85.64 ± 5.84	0.331
Heart rate	91.35 ± 16.04	94.36 ± 18.16	0.487
Respiratory rate	23.54 ± 4.32	24.73 ± 5.17	0.321
Leucocytes	9.35 ± 4.97	12.37 ± 3.27	0.012
Neutrophils	8.05 ± 4.91	11.13 ± 3.20	0.009
Lymphocytes	0.93 ± 0.52	0.70 ± 0.42	0.075
Glucose	137.43 ± 73.57	184.40 ± 144.84	0.163
ALT	66.01 ± 95.00	43.04 ± 38.63	0.327
AST	73.73 ± 148.66	51.39 ± 58.29	0.529
LDH	487.88 ± 327.41	505.31 ± 182.31	0.844
Total bilirubin	0.61 ± 0.38	0.69 ± 0.27	0.474
CK	105.65 ± 111.02	122.15 ± 84.85	0.706
Glomerular filtration rate	94.80 ± 25.50	75.10 ± 35.79	0.030
Fibrinogen	1513 ± 2803.29	862.80 ± 453.92	0.617
PCR	18.04 ± 10.23	26.68 ± 13.16	0.012

## Data Availability

The data used to support the study are available from the corresponding author upon request.
